# “The Panhandle is Different than the Peninsula”: How Rural Colleges in Florida Implemented Education Reform*

**DOI:** 10.1111/ruso.12309

**Published:** 2019-11-06

**Authors:** Amanda N. Nix, Tamara Bertrand Jones, Shouping Hu

**Affiliations:** Florida State University

## Abstract

When it comes to the creation of higher education policy, state legislators are challenged with addressing the diverse academic needs of college students enrolled across a spectrum of institutions. In this paper, we explore how 203 individuals at five rural-serving colleges in Florida engaged in state-wide developmental education (DE) reform using the theoretical framework of “situated cognition.” Specifically, we ask whether the colleges implemented DE reform in unique ways that may have differed from their non-rural counterparts and what their rationale was for doing so. Our work indicates that institutional culture and capacity generated a combination of strengths and constraints in how reform played out in a rural context. In some ways, a rural-serving identity made colleges more flexible and better able to adjust their advising and curricular structures to comply with the mandates of SB 1720. But in other ways, rural-serving institutions lacked the resources necessary to adequately support students and campus personnel through this dramatic transition toward new ways of providing DE. In looking toward the passage and implementation of future education reform, we call upon policy-makers to consider more deeply the design of policies to ensure they support rural and non-rural colleges alike.

## Introduction

The state of Florida, home to approximately 21 million people, is America’s third most populous state. From Pensacola to Key West, a diverse group of individuals call the state home. According to the United States Census Bureau (2017), 20 percent of the state’s population is foreign-born and 20 percent are aged 65 or older. In addition, there are a number of distinct regions throughout the state, like the Treasure and Space Coasts, which reflect the unique industries and cultures of local populations. Despite an estimated 11.6 percent growth in statewide population between 2010 and 2017, there are also certain places that remain very rural, particularly in the panhandle and areas to the west of Lake Okeechobee (Florida Department of Health n.d.).

When it comes to the creation of social policy, state legislators are challenged with simultaneously addressing the needs of these myriad groups and geographic regions. Higher education is one place where consistent implementation of statewide social policy can be difficult. We focus this paper on developmental education (DE) reform, namely Florida Senate Bill (SB) 1720, and its implementation throughout the state by rural-serving colleges.

SB 1720 was passed in the spring of 2013. It notably made college placement testing and, by extension, DE courses optional for “exempt” students (defined as having entered a Florida public school as a ninth grader in 2003–2004 or after, and graduating with a standard Florida high school diploma or holding active duty military status) in the Florida College System (FCS). According to the law, students who chose to enroll in optional, remedial-level math, English, or reading would be provided redesigned courses “delivered through a variety of accelerated and corequisite strategies” (SB 1720 2013:27). In order to make reform efforts more successful, SB 1720 also called upon colleges to increase their emphasis on academic advising and student support services.

In view of how different FCS institutions are from one another, especially due to geographic context, we explore how rural-serving colleges implemented state-wide DE reform between 2014 and 2018. Specifically, we ask whether rural-serving colleges implemented DE reform in unique ways that may have differed from their non-rural counterparts. If so, what was the rationale for them to do so? To answer these questions, we employ the theoretical framework of situated cognition (Spillane, Reiser, and Reimer 2002). What we find is that institutional culture and capacity both played major roles in how rural colleges in Florida revised their curriculum, provided advising services, and increased available academic supports following the passage of SB 1720.

Presently, community colleges do not receive near the same attention as four-year colleges and universities in published scholarship (Levin and Kater 2013). This inequity is even more dramatic for community colleges located in rural areas (Sipple and Brent 2009). Lack of interest carries over into politics as well. The welfare of rural-serving colleges has long been overlooked by legislators when policy is enacted (Katsinas and Hardy 2012; Sipple and Brent 2009). This oversight occurs to the detriment of many students, considering one-third of those who attend a community college attend a rural-serving institution (Katsinas and Hardy 2012). There has been some renewed interest in rural-serving colleges in recent years, but there is much left to be said about them, particularly related to the importance of DE within rural communities (Koricich and Boylan 2019) and the impact of education reform (Smith 2019). Our work serves to address the gap in scholarship by highlighting the experiences, challenges, and strengths of rural-serving institutions across Florida as they attempted to implement DE reform.

## Theoretical Framework

Education reform is often passed via state or federal action, and is then carried out by local environments with a great deal of autonomy and discretion. According to Spillane and colleagues (2002), this autonomy and discretion ultimately shapes what reform looks like across different locations. The resulting variation can be explained by the scholars’ cognitive framework, which contends that individuals interpret and make sense of social policies according to three things: their existing cognitive structures, their situational context, and the policy signals they receive.

The first dimension of the framework—individual cognition—highlights the importance of an individual’s knowledge, beliefs, and attitudes when interpreting and implementing policies. The second dimension, which is the primary focus of our research, considers the multidimensional impact of one’s situational context, including “social, material, intellectual, temporal, historical, and cultural aspects” (Spillane et al. 2002:412). In other words, situated cognition acknowledges that institutions—in this case colleges—and their employees have social networks, values, personal and organizational histories, and so forth, which influence understandings of reform. The third dimension of the framework is dedicated to policy signals, encompassing policymakers’ intentions, the text of the legislation itself, and implementers’ understanding of it. Ultimately, it is this three-part “social sense-making process” that guides individuals and campuses as they revise and adapt legislated policies, in many cases unconsciously, to better suit their unique circumstances (Spillane et al. 2002).

This theoretical approach to education reform shares much in common with the study of cognitive sociology, which calls attention to the ways in which “thought communities” (e.g., religions, professions, and countries) influence how individuals think about the world around them. In the words of Zerubavel (1997:6), “we think not only as individuals and as human beings, but also as social beings”. As such, thinking, even about topics of reform implementation, is shaped by our social environment and cognitive interactions with the world.

SB 1720 was primarily a framework for educational reform, granting colleges a good deal of latitude throughout the implementation process. Here, we consider how the situational context of rural-serving colleges shaped the actions taken by campus personnel related to advising, academic support services, and curricular redesign.

## Literature Review

Rural areas, including those in Florida, are commonly defined by elevated levels of poverty, food insecurity, and unemployment compared with more urban areas (Economic Research Service 2018). These social problems date back to the 1970s and 1980s, and have continued to grow in severity since (Elder and Conger 2014).

### Trends in Rural Postsecondary Education

Research tells a similarly discouraging story about postsecondary education outcomes in rural areas. These communities generally face low levels of college attendance and bachelor’s degree attainment (Hu 2003; Koricich, Chen, and Hughes 2018; Provasnik et al. 2007). Those who do attend college often exhibit different attendance patterns than their urban and suburban peers; rural students commonly delay entry to college, have breaks in enrollment, and attend less selective institutions overall (Byun, Irvin, and Meece 2012; 2015). One explanation offered by scholars is that fewer academic and financial resources are made available to students in rural areas through families and schools, leading to rural deprivation (Roscigno and Crowley 2001; Roscigno, TomaskovicDevey, and Crowley 2006). Others point to barriers, like marriage and familial responsibilities, which play a unique role in the lives of rural youth (Irvin et al. 2012).

Some scholars, moving away from a deficit perspective, highlight the advantages of rural life for students interested in postsecondary education. These scholars emphasize the numerous social resources available because of the tightly knit, and often overlapping, connections fostered among families, schools, churches, and community groups (Crockett, Shanahan, and Jackson-Newsom 2000; Elder and Conger 2014). Indeed, life in a rural community has been associated with improved chances for college attendance when socioeconomic status is controlled for in statistical models (Byun, Meece, and Irvin 2012; Perna 2000). However, due to the high rates of poverty in rural areas, levels of postsecondary attendance and achievement remain low.

An ongoing discussion in the literature also highlights the importance of one’s geographic proximity to a college or university for education outcomes. Past findings indicate that proximity is a critical component in the college choice process (Turley 2009), particularly for those in rural communities where family ties are prioritized and financial resources are limited (Johnson, Elder, and Stern 2005). Unfortunately, not all students have the same access to rich postsecondary opportunities, resulting in education deserts (Hillman 2016).

### The Importance of Rural-Serving Colleges

Rural-serving community colleges play a critical role in remedying these deserts by expanding access to higher education in hard-to-reach areas (Koricich et al. 2018). To this point, students living in rural areas more commonly aspire to (Hu 2003) and attend two-year institutions (Bowen, Chingos, and McPherson 2009; Koricich et al. 2018), as compared to their urban and suburban peers. This is likely because of the accessible and convenient location of community colleges. In Florida specifically, many of the FCS institutions operate in areas that are geographically removed from other private colleges and State University System institutions.

Collectively, rural-serving colleges enroll more than one-third of public community college students. During the 2007–2008 academic school year, this equated to approximately 3.5 million students enrolled at 575 two-year public college campuses nationwide (Katsinas and Hardy 2012). Rural colleges also benefit the wider community by functioning as engines for local economic development, hubs for cultural enrichment, centers of learning and workforce development, and employers themselves (Cavan 1995; Crookston and Hooks 2012; Siegfried, Sanderson, and McHenry 2007). In some communities, their impact extends even further to include the development of community inclusiveness, community pride, and a value-added lifestyle (Miller and Tuttle 2007).

### The Challenges Facing Rural-Serving Colleges

Because of their unique student population, education on rural-serving community college campuses looks different than it would elsewhere. Compared to their suburban and urban counterparts, rural-serving institutions are comprised of more full-time and residential students. They are also less racially and ethnically diverse (Hardy and Katsinas 2007). Furthermore, colleges located in rural areas often serve students with personal circumstances (e.g., limited transportation and childcare or elder care responsibilities) that can present barriers to academic success (Gillett-Karam 1995). In light of this, DE programs play a critical role for rural-serving colleges and their students (Koricich and Boylan 2019).

In addition to helping students manage and minimize the impact of personal issues, colleges themselves are faced with a number of challenges unique to their identity and culture as rural-serving institutions. To begin, there is the challenge of providing a comprehensive curriculum (Cavan 1995), despite limited enrollment and the inability to leverage economies-of-scale (Hardy and Katsinas 2007; Pennington, Williams, and Karvonen 2006). In addition, rural-serving colleges struggle with an aging student population, financial constraints (e.g., funding inequities and difficulty obtaining grant dollars), and finding qualified job applicants for teaching and administration (Pennington et al. 2006).

Policymakers do not always consider these challenges in the creation and passage of education policy (Katsinas and Hardy 2012; Sipple and Brent 2009). The allocation of state funds based on enrollment numbers, for instance, is highly problematic for rural institutions since they typically enroll fewer students than suburban and urban colleges (Pennington et al. 2006). Similarly, performance-based funding measures can disadvantage small, rural colleges due to low student enrollment and limited staff resources (Thornton and Friedel 2016).

In the 1980s, the state of Florida was concerned with the unintended consequences of “sincere effort to upgrade the entire educational system” on small/rural community colleges (Phillips 1983:58). The passage of new legislation related to high school competency tests and college-level achievement tests, among other things, generated unique challenges for those in rural communities, particularly related to funding, teacher recruitment/retention, and program comprehensiveness. In the findings that follow, we raise similar concerns about the consequences of more contemporary legislation—SB 1720—for Florida’s rural-serving colleges, but also highlight the advantages these institutions were able to leverage when implementing reform efforts.

## Methods

Here, we present a multiple-case study of five, rural-serving state colleges in Florida. In accordance with multiple-case studies, our research process involved selecting the five cases, conducting site visits, writing individual case reports, and drawing cross-case conclusions (Yin 2013). Based on those conclusions, we were then able to make connections to situated cognition and develop implications for policy.

### Context of the Study

According to the Florida Department of Health (n.d.) and 2010 U.S. Census data, 30 counties in the State of Florida qualify as rural, having a population density of less than 100 people per square mile. Individuals living in these 30 counties are served by 13 of the 28 FCS institutions. The documented diversity of rural-serving colleges (Koricich 2012), is apparent among these 13 FCS institutions and is worth noting. For one, the makeup of the student body varies dramatically from college to college. Some rural-serving colleges located in South Florida have high percentages of Latinx students (Florida SouthWestern State College n.d.), while others in the panhandle have large concentrations of active duty and veteran students due to their proximity to military installations. In many cases, the courses and programs offered by the schools vary accordingly, reflecting both the composition of the student body and employment needs of the surrounding community. As an example, Florida Keys Community College is home to a hyperbaric chamber for those training as diving medical technicians (Florida Keys Community College n.d.) and the College of Central Florida operates Vintage Farm for those in the Equine Studies and Agribusiness programs (College of Central Florida n.d.).

Also, the colleges vary in size. The 2010 Basic Carnegie Classifications of Institutions divides rural-serving community colleges into the following categories: small (<2,500 annual unduplicated head count), medium (2,500–7,500 annual unduplicated headcount), and large (>7,500 annual unduplicated headcount) (Carnegie Foundation for the Advancement of Teaching 2010). In total, the FCS served 801,023 students during the 2015–2016 academic school year. Some of the colleges located in Florida’s most rural areas, such as Chipola College, the Florida Keys Community College, and North Florida College, saw enrollments of fewer than 3,500 students. Other colleges that serve rural counties, like Tallahassee Community College and Indian River State College, enrolled more than 25,000 students (Florida Department of Education n.d.).

This research highlights a subset of data pulled from a larger research project examining implementation of SB 1720 on 21 of the 28 FCS institutions. We chose to restrict our sample in accordance with the Carnegie Foundation for the Advancement of Teaching’s (2010) definition of public, rural-serving “Associate’s Colleges”:

Urban-serving and suburban-serving institutions are physically located within Primary Metropolitan Statistical Areas (PMSAs) or Metropolitan Statistical Areas (MSAs), respectively, with populations exceeding 500,000 people according to the 2000 Census. Institutions in PMSAs or MSAs with a lower total population, or not in a PMSA or MSA, were classified as rural-serving.

We also supplemented formal Carnegie classifications with 2010 U.S. Census data (Florida Department of Health n.d.) and self-identification; individuals at all five participating colleges emphasized a rural identity in their response to focus group and interview questions. In terms of enrollment size, we present the perspectives of those at institutions falling into all three of the previously defined categories of small, medium, and large based on 2017 enrollment figures. That being said, even the largest college included in our sample is small relative to the enrollments of urban FCS institutions like Valencia, Broward, and Miami Dade College.

### Data Collection

Data collection began with initial site visits to five rural-serving colleges between 2014 and 2017. We returned to four of the colleges once more between 2016 and 2018 at their convenience. During the follow-up visits, we spoke with new and repeat participants about their perceptions of rural identity and policy implementation.

Site visits lasted one to two days and involved a small team of two or more researchers. The institutions themselves made all necessary arrangements for the visits, involving scheduling, logistics, and the recruitment of participants from various contingents (administrators, faculty, advisors, other support staff, and students) of campus. At some colleges, we provided incentives in the form of gift cards or catered lunch to specifically encourage student participation.

As shown in Table 1, we report perspectives expressed by 203 individuals during 36 semi-structured focus group sessions and 4 individual interviews. In focus group sessions, participants were asked questions based on their status as administrators, faculty members, advisors, or students. Administrators were asked questions about such topics as the decision to offer certain DE courses and modalities instead of others, the role of technology on campus, and the impact of SB 1720 on students and personnel. Faculty and advisors had the opportunity to share their perspectives on topics like cross-campus collaboration, changes to advising and instruction, and the growing role of student support services. Students were asked about career goals, the decision to attend their respective college, and challenges they had faced or supports they had received in pursuit of higher education. On one site visit, interviews were arranged in lieu of focus groups because only two administrators were available to participate. However, the same protocol was used with focus group participants and interviewees alike. Separately, we interviewed two college presidents and asked, among other topics, about unanticipated barriers or constraints to implementing SB 1720 at their institution. The presidents expressed unique perspectives as the leaders of rural-serving institutions, which provided context for interpreting data collected during campus visits.

**Table 1:** Summary Count of Participants.

	Size	No. of Visits	Focus Groups	Interviews	Participants
College 1	Small	2	8	1 Presidential interview	48
College 2	Medium	2	8	–	50
College 3	Medium	2	3	2 Administrator	15
				interviews	
College 4	Large	2	14	1 Presidential interview	76
College 5	Large	1	3	–	14
Total		9	36	4 Interviews	203

In addition to focus group and interview data, our findings were also informed by a number of other sources. These included college websites, site visit observations, and documents (e.g., SB 1720 implementation plans and accountability reports) submitted by the colleges to the Division of Florida Colleges. While we do not explicitly reference these sources in our findings, they were instrumental in helping us to identify and clarify our thinking around important patterns and themes related to campus culture, course offerings, and institutional finances.

### Data Analysis

The first cycle of coding consisted of a research team engaged in descriptive, process, and emotion coding (Saldaña 2009). These different approaches to data analysis were complementary, allowing us to document both the actions taken by individuals to implement SB 1720 and their reactions—positive and negative—to the bill. When appropriate, we also completed simultaneous coding, a method whereby paragraphs of text were assigned a number of appropriate codes at the same time to reflect the interconnected nature of the data (Saldaña 2009). It was during this first round of coding that rural identity first emerged as a noteworthy construct and we began to formulate an understanding of how rurality might have shaped policy implementation.

During second cycle coding, we narrowed our focus to institutional culture and capacity. The process involved combining, deleting, and renaming codes to better reflect the patterns that previously emerged in first cycle coding. Using situated cognition as a guide, we paid special attention to instances where participants spotlighted their institution’s rural identity by comparing implementation of SB 1720 on their campus to how they perceived it was implemented on urban or suburban campuses. Ultimately, the findings we present come from several codes: *campus culture, collaboration and coordination, consequences of the legislation, perceptions of access to technology, challenges to student learning*, and *enrollment*.

Each year between 2014 and 2018, the research team completed member checks with participating institutions to confirm the accuracy and clarity of our interpretations drawn during site visits and data analysis. Researchers also discussed important findings with peers knowledgeable about community colleges and DE reform, providing the chance for them to question our interpretations as devil’s advocates, or critical friends (Miles, Huberman, and Saldaña 2014; Patton 2015).

In the section that follows, we present findings that emerged through this analysis process. We emphasize that the institutional culture of rural-serving FCS institutions, characterized by relationships, collaboration, and support, provided a number of advantages during implementation of SB 1720. In contrast, limited capacity, particularly related to enrollment, finances, and technology, presented some challenges to reform. We also use the space to draw important connections between our findings and previous research conducted by others in the field. This form of organization allows us to fully interrogate the significance of situated cognition for the implementation of SB 1720 at rural-serving institutions, one key theme at a time.

## Findings

Even without being asked, participants readily acknowledged the unique and rural identities of their individual colleges, using phrases like “cow country” and “the sticks” to describe their service area. They also commonly referenced student enrollment, reiterating an identity as “small” colleges, particularly in comparison to their urban counterparts in cities like Ft. Lauderdale and Miami. Notably, rural identity was a point of pride for many participants including one administrator who boasted: “We are small…, small but mighty.”

Several campus personnel highlighted how the institutional differences generated by geographic context clearly intersected with the legislation. According to one administrator, “The panhandle is different than the peninsula… When decisions are made, we like to think they are made considering all the state of Florida… [including] the more rural situations that do not fit the same [mold] as Dade or Broward.” In the case of SB 1720, he was concerned that the well-being of “all the students across the state” had not been considered. According to another participant, policymakers should not have expected that “one shoe fits all,” when the service areas of even one institution includes a combination of rural communities, military installments, and beach-front communities. This challenge is more acute when considering the entire state of Florida and the diversity that comprises it.

In the face of such complexity, one advisor reflected upon the fact that the legislation should have provided some exemptions for small, rural colleges that cannot implement reform in the same ways as large institutions: “I just don’t think that there is one answer that’s uniform for this state… [or] our [multiple] locations… We have got to have exemptions from some rules, and policies, at these outlying centers.”

Upon further inquiry, we determined that these sentiments arose from campus personnel’s fear that SB 1720 would decrease student success and undermine institutional efforts to foster equity among different student groups. In the words of an administrator, “We have to be mindful of these broad stroke changes that we make in education because things that work in some places will not work in others, and equity is, maybe, being compromised.” In many cases, campus personnel thought that some of the provisions of SB 1720 were challenging to implement in a rural environment, putting low-income students and other groups at risk of academic failure.

Despite these initial concerns, when it came to implementing the reform rural-serving college administrators expressed a compliant attitude: “We’re compliant people… We all work so hard and have so much to do on a small campus… and the students have so many needs and so there’s not a lot of extra time to spend resisting anything.” Instead of resisting, campus personnel at these rural institutions implemented the provisions of SB 1720 as best they could according to their understanding of “the spirit of it.” This finding supports Spillane and colleague’s (2002) assertion that individuals doing the day-to-day work of implementation do not commonly undermine or sabotage efforts at change.

According to these data, we find that those at rural-serving institutions interpreted the spirit of SB 1720 and implemented its provisions in ways that made sense to them based on the material, social, cultural, and other aspects of their situation (Spillane et al. 2002). In the sections that follow, we describe how institutional culture and capacity each played a role in the cognitive behavior undertaken by campus personnel during reform.

### Institutional Culture: “The President… Knows Everybody”

SB 1720 was implemented on rural-serving college campuses in particular ways because of pervasive institutional culture expressed in the form of relationships, collaboration, and student support. Together, these findings speak to the social and cultural aspects of “situation” highlighted by Spillane and colleagues (2002) in their framework. Interestingly, all three cultural elements elaborated upon here were found to be beneficial, allowing rural-serving campuses to easily and successfully implement the provisions of SB 1720.

#### A culture of relationships

Participants described their campus culture in a variety of ways, but consistently emphasized the “tight knit” and relational nature of faculty, staff, and student interactions. As an administrator summarized, “I love [working for my college]… Our students… know the faculty and the staff who work here. Everybody cares about them as individuals.” At one college, even the president engages in building this culture. According to students:

Student 1: [Our college] is small, so… it’s no strange thing for the president of the college to be sitting out here on the bench and see a student walking by and say, ‘Hey, get over here for a minute. I want to talk to you…’ How many times has he caught one of you guys and he made you sit down and talk to him…?

Student 2: All the time.

Academic advising was one functional area where a culture of relationships was overtly expressed. Because of the complexity of reform mandates, SB 1720 required that all first time in college students participate in academic advising. After the initial advising session, many students chose to return to their advisors for additional guidance. Several advisors detailed this strong student preference for face-to-face interactions over online registration, even when presented with long wait times. We believe that this express desire for relationships with campus personnel was rooted in the values and culture of rural life to which students are accustomed (Elder and Conger 2014). Fortunately, research suggests that the additional investment required to cultivate these kinds of “responsive relationships” generates benefits, promoting increased retention at rural community colleges (Howley, Chavis, and Kester 2013).

Many colleges also made the choice to engage advisors in newly designed early alert systems designed to catch students who might be struggling personally or academically, particularly in the wake of SB 1720. When presented with a list of students at risk of academic failure, advisors at one college described going to great lengths to track down their assigned students. One noted,

If they do not return the phone call—and I know everyone in here has done this—if we find out a class they’re going to, we will go to that class and wait for them to come out and say, ‘I need to see you.’

Another added that advisors will also approach students off-campus, at places like the local Walmart, to offer additional academic support. When asked about these practices two years later during a follow-up visit, campus personnel confirmed that the trend persisted. Advisors at the other four colleges similarly spoke about using a combination of phone calls, emails, and Facebook messages to make contact with hard-to-reach students. Such efforts clearly demonstrate a culture of relationships lived out by the students and personnel at rural-serving institutions. This level of familiarity and intimacy simply would not be possible on a larger, more urban campus.

Participants shared that this culture of relationships extended beyond the advisor–student dynamic to include faculty, staff, and upper level administration. According to one advisor, even the president of the college prioritizes relationship building:

All of my first-time advising sessions [begin] with, ‘I’m glad you’re here. You have got to get to know your professors… They’re the best. They have office hours [and] they’ll make time after office hours or to fit your schedule. Whenever you think that you’re missing a concept or you’re falling behind, just make an appointment… They’re available. They’re here. They’re friendly… The president of the college knows everybody that’s on campus.’

Faculty and advisors also reported strong relationships with one another. Indeed, one advisor noted this as a strength of small, rural institutions: “I think that’s some of the benefits of a small school… We all know the faculty and they feel comfortable with talking with us about students, particularly when it comes to the developmental ed.” The benefits of these relationships between campus personnel were two-fold; not only did campus personnel enjoy meaningful and positive interactions with one another but their students, especially those put at risk by the legislation, profited from the extra support and encouragement the interactions generated as well.

#### A culture of collaboration

Rural-serving institutions also implemented SB 1720 in certain ways due to a culture of campus-wide collaboration. In many cases, this meant that individuals across campuses were involved in sharing opinions and ideas about implementation before a final plan was put into place. These interactions are a clear example of the social aspect of sense-making emphasized by Spillane and colleagues (2002). As one administrator described,

Everyone kind of came together and said, ‘Well what about this?’ And we kind of unpacked each part. What does a corequisite look like? … And we thought, ‘Well as faculty that would be a great idea.’ So [the registrar]… would say, ‘All right. How would that look on [our Enterprise Resource Planning system]? What if they pass one and don’t pass another? How do we register them for both?’… We were constantly meeting, and updating, and disseminating information. It was a good group effort.

Another administrator at the same college confirmed that implementation of SB 1720 was “a school-wide initiative…, all hands on deck.” These sentiments shine light on the reality that rural-serving colleges often need campus personnel to fill a number of administrative functions (Hardy and Katsinas 2007). This finding also speaks to the previously defined culture of relationships evident on rural campuses. Because individuals saw themselves as a team dedicated to student success, they were able to work together to enact grassroots change.

SB 1720 presented Florida colleges with many decisions that needed to be made in a short period of time. Rural-serving colleges are known for the ability to implement change quickly (Hardy and Katsinas 2007; Smith 2013), and the implementation of SB 1720 proved to be no exception. The culture of collaboration described here served rural colleges well throughout implementation, allowing campus personnel to rapidly implement change. To this point, an administrator noted:

In terms of Senate Bill 1720, the fact that we were so nimble and flexible [was beneficial]. I think when you look at a [large, urban] college… Turn[ing] that ship in a different direction takes a lot of time. For us, you just get the folks in the room and you’re like, ‘Okay, this is what we have got to do. How are we going to do it?’

Another administrator highlighted the fact that decisions regarding SB 1720 were made quickly and easily because offices and departments were in close proximity to one another: “We are small. We are physically located close together and so many decisions are made as they need to be made.” In a larger, more bureaucratic environment, change would certainly have been slower and more cumbersome.

Colleges engaged in both internal and external collaboration. In several cases, campus personnel reported working with community members to design their local implementation of SB 1720. According to one administrator, his college invited two district representatives to sit on the implementation committee and also solicited feedback from high school instructors. An advisor at another college called attention to their work with area high school staff to set and clarify student expectations about college enrollment, attendance, and performance. These noteworthy collaborations with the local community have allowed the colleges to better anticipate and attend to the difficulties students might face in the transition from high school to college, especially related to the implementation of SB 1720.

#### A culture of support

In addition to culture characterized by relationships and collaboration, participants also described a culture of support. Indeed, campus personnel spoke repeatedly about their concerns with the provisions of SB 1720 and the impact they might have on the success of rural students. This genuine concern for student well-being was rooted in longstanding ties between the college and community and an intimate familiarity with current levels of student preparation. For one administrator, implementation of SB 1720 is “a process that we’re working through.” She added, “We live in the districts ourselves, and so we know the people we go to school with and go to church with and call into our office. So, we’re invested in these students.” Another administrator described a similar reality: “We know our district students better than anybody else and we know their strengths and their weaknesses. And so our own children, we send them here and I think that says a lot for what our campus does for the community.”

Regarding academic weaknesses, campus personnel were concerned that rural students needed additional support to mitigate the risks of SB 1720 because of their disadvantaged academic backgrounds. Participants made numerous connections between rural identity, poverty, and first-generation status. A faculty member noted, “We just have an extraordinary number of students who have don’t have two extra pennies.” Because of this, an administrator shared that many of the students attending her school were severely underprepared according to high school placement test scores: “One of our counties, we had 84 seniors last year take the PERT test in high school. Two passed enough to get into college classes… That’s where some of these kids came from.” Considering these statistics, campus personnel felt many students needed increased academic supports in order to be successful at the college level. To this same point, an advisor added: “One thing we see is a lot of our students are coming from small high schools. They don’t have the study skills or the note taking skills.” Here, the under preparation of some rural students extended beyond specific course content to more general, “college success” skills.

Colleges addressed these challenges with academic preparation, made all the more evident by SB 1720 and optional DE courses, by reorganizing and expanding academic support services. Some colleges extended tutoring coverage to support courses like Intermediate Algebra and Calculus 1 that were previously excluded. Others moved staff into larger facilities to better handle the increased demand for services. According to one administrator, “Our math lab was only one-classroom sized at the beginning. Now it’s taking up two. It had to expand, and that’s the same thing with the writing and reading lab.” Additionally, several colleges “commandeered faculty” and required them to hold office hours and meetings in spaces managed by academic support services to get students more comfortable with dropping by.

In general, academic support services are underutilized throughout the FCS (Hu et al. 2016a). However, we find that tutoring, writing centers, and labs played an essential role on rural-serving campuses following the implementation of SB 1720. Even students acknowledged their value: “I went to the tutoring [center] and they’re very helpful… They’ll walk you through the problem… If you take a quiz, they’ll go over it with you and tell you if you did it wrong.” We posit academic supports were perceived as successful in these environments because of the relational nature of campus life at rural-serving institutions.

Participants also noted that academic support services were particularly effective because they had enough personnel to serve the student body. One administrator pointed out that, due to her college’s enrollment size, “You can do a lot of one-on-one tutoring with that small of a group… They really go all out to get the students there and involved.” This is a strength unique to rural-serving colleges; large urban institutions would likely need to maintain higher student-to-staff ratios.

### Institutional Capacity: “We Can’t Build Classes of an Adequate Size”

In addition to culture, implementation of SB 1720 played out as it did on rural-serving campuses because of constraints imposed by limited institutional capacity (i.e., the material component of “situation”). In the words of Spillane et al. (2002:390), “implementing agents and agencies also often lack the capacity—the knowledge, skills, personnel, and other resources—necessary to work in ways that are consistent with policy”. Indeed, according to participants, some elements of DE reform were described as simply incompatible with the day-to-day realities of life at a rural-serving institution due to cost, enrollment, or infrastructure. Even still, campuses complied to the best of their ability.

#### Capacity to redesign coursework

Rural-serving colleges struggle to provide sufficient course offerings to their students. Out of practicality, for instance, they offer fewer classes on weekends than other colleges (Hardy and Katsinas 2007). This constraint was worsened by the provisions of SB 1720, which made DE courses optional for some. According to two administrators, DE enrollment is now especially low:

Administrator 1: We don’t have that many DE students.

Administrator 2: Yes, very few. I mean four in one semester, that’s it, and that’s for reading and writing. Two for reading, two for writing.

Administrator 1: When we only have one or two students, it [the corequisite DE component] becomes an independent study. Finding people to staff the independent studies is sometimes difficult.

Furthermore, SB 1720 required that colleges offer students the choice of DE course taught in a compressed, modularized, contextualized, and/ or corequisite format. In some cases, however, rural colleges struggled with finding sufficient enrollment to offer one, optional DE course, much less several of them taught in different ways. For this reason, an administrator pointed out that enrollment numbers were simply too low to successfully implement an adequate variety of redesigned courses, as described by legislators in the language of the bill: “There, again, you’re talking to a small [college]. I cannot imagine that working here… We might could do a dev. ed. for nursing because we have a lot of nursing students. But otherwise… manpower would be very difficult.”

Notably, these challenges were not exclusive to small or medium sized colleges. Because rural-serving institutions have expansive service areas, student enrollment often spreads across many satellite campuses. In light of this, an administrator at a large, rural-serving college expressed concerned for students needing to take DE reading somewhere other than the main campus: “Our challenge there is enrollment, and getting sufficient students, especially in a [DE] reading course to make… I mean, if I got three students, what am I going to do?” To this, she added:

I can’t ask a faculty member to go up there and pay per head for three students… My budget is tight… Or I can’t say to the student, ‘Nope, you can’t come to college because of that reading course’… And a lot of our students, you know, you can’t say to the student in [in an outlying county], who hasn’t got a car, ‘Well, just drive to [the main campus], we’re running it here.’

An interesting point also arises here about the intersection of challenges inherent to rural life that generate cumulative disadvantage for students in terms of accessing a diverse educational curriculum full of choices. In an effort to simultaneously solve the enrollment and transportation problems, one campus offered developmental-level reading and writing courses on one satellite campus and a math course on another, with the plan of switching the available courses in subsequent semesters. Nevertheless, an administrator reported, “It still didn’t work; we can’t build classes of an adequate size.”

#### Technological capacity

In addition to enrollment numbers, rural-serving colleges struggled to implement redesigned courses taught via certain modalities due to a lack of information and communications technology (ICT) infrastructure. Modularized DE courses, in particular, are taught using instructional software. However, rural-serving institutions and their surrounding communities do not receive the same investment in technology and online connectivity as urban communities. For one, rural communities and the institutions that serve them have long faced limited access to technology, captured by metrics like computer penetration, E-mail usage, and availability of high speed Internet (Katsinas and Moeck 2002; Salemink, Strijker, and Bosworth 2017). Reflecting this traditional conception of the “digital divide,” one advisor explained, “This is a rural area. Many of them don’t have computers, don’t have access to computers.” For this reason, some students sit in the parking lots of businesses that offer free wireless Internet to complete DE classwork, or do online homework on their smart phones. Neither option is ideal for students who are already facing a number of other barriers to academic success. According to one faculty member, “One lady that I had was a good student. She’s got her phone, but she said when she pulls up the exercises… it’s so small that she hits the wrong thing… So, they [rural students] are at a disadvantage.”

While individuals continue to struggle with access, as demonstrated above, the contemporary US digital divide is now characterized by more nuanced issues of *technology maintenance,* or the ability to consistently and reliability access technology (Gonzales 2015). Research shows that the quality of data infrastructure in rural areas is lower than that in urban areas (Salemink et al. 2017). Illustrating this challenge, one participant highlighted how the instability of ICT infrastructure in the surrounding community impedes academic pursuits: “If they do have a computer, being rural, it’s not always working. [Laughs] Your Internet doesn’t always want to work that day. Your satellite link doesn’t [either].” Limited or unreliable access to technology represent examples of the material dimension of “situation,” included by Spillane and colleagues (2002) in their definition.

An advisor, acknowledging the fact that “most of our service area is a pretty rural area,” highlighted a third component of the digital divide— disparities in skill and comfort with using technology (Hargittai 2002; Salemink et al. 2017): “Whether it’s socioeconomic status or educational level of their parents or whatever, they’re just like, ‘I don’t want to touch one of them computers.’” Another advisor added that many of her students “don’t know how to turn a computer on.”

Interestingly, Pennington and colleagues (2006) found in their study of rural-serving community colleges in Kansas that campus personnel were excited about the role technology might play in extending their service area into hard-to-reach communities. Faculty involved in our study similarly acknowledged the value of computer-based learning:

It has just so many little explanations to where if they don’t know how to do a problem, it will walk them through, step-by-step, and have them put in an answer to each little step. But then, once they get through that, it requires them to do a similar problem… And in my experience, it’s been a pretty good tool for them to use.

However, there have been notable challenges for rural-serving Florida colleges as they redesigned DE courses due to the digital divide. In response, institutions report having increased the number of on-campus, technological resources available to students so that they can complete their modularized, hybrid, and other computer-based DE coursework. This represents another example of how reform efforts were unique to certain kinds of institutions based on their situational context.

#### Financial capacity

One commonly cited challenge faced by rural institutions nationwide is a lack of adequate funding (Pennington et al. 2006). In support of this, many participants noted that campus resources were strained prior to the passage of SB 1720. Unfortunately, SB 1720 unintentionally “exacerbated some of the [financial] issues we were dealing with” by requiring DE reform without any provision of additional funds for implementation. In other words, SB 1720 was passed as an unfunded mandate that required schools to extensively redesign DE curriculum and implement certain practices, like increased advising services, without any state dollars allocated to these efforts.

In order to bring their rural-serving colleges into compliance, campus personnel had to find ways to do more with less. For many, this involved completing more tasks, serving more students, and working longer hours. In the words of one administrator, advising has become particularly time consuming and labor intensive:

We have a small advising staff… We essentially have two individuals, who are supported by others, who take on some of this work. When you move from an admission process that was… somewhat automated because you had the placement test that was clear-cut… to one that is more subjective, where you’re looking at a student’s transcript… that takes a whole lot longer.

Accordingly, a president at a rural-serving institution revealed that implementation of SB 1720 “has been quite taxing… For an institution like ours, where we don’t have a lot of people in the advising area, that’s been really significant. It’s been considerable.” Colleges have also been confronted with the reality of having limited funds to fill the educational and programmatic gaps left behind by a shrinking DE program. Unfortunately, one administrator noted that, “With this being a small school, we could not really do some of the individual programs that the larger schools do. We just didn’t have enough students to make that a viable option.”

Rural-serving colleges were able to successfully implement SB 1720 in the face of financial constraints due, primarily, to the culture of relationships previously described. Campus personnel expressed feelings of loyalty to their college, even when asked to work overtime or take on new responsibilities without a corresponding pay raise. To this point, one administrator acknowledged, not only the extra work being done by faculty and staff, but also the motivating factor behind it—dedication to the student body:

There were challenges that came with this [SB 1720] that we worked hard to address. I mean it is what it is and we have to move forward and do what’s best for students and we’ve tried to do that with no extra resources. We are asking more, I think, of faculty with no compensation. And because we’re small and they’re dedicated to the students and the community they’re willing to do that.

SB 1720 has required more work of people at institutions across the state (Hu et al. 2016a), but these data indicate that the challenge is particularly significant for those on rural-serving campuses who were already stretched thin, even before the bill passed.

## Conclusion

Spillane and colleagues (2002) present a framework of situated cognition that emphasizes how people implement one policy in many different ways due to their existing cognitive structures, their situational context, and the policy signals they receive. SB 1720 presented the FCS with dramatic change, much like the standards based reform that informed the creation of the framework. We similarly find that five colleges in Florida implemented SB 1720 in particular ways due to their rural “situation.” More specifically, institutional culture and capacity played significant roles in how the colleges redesigned their curriculum, provided advising services, and increased available academic supports following the passage of SB 1720.

Interestingly, our work reveals that institutional culture and capacity generated a combination of advantages and constraints during reform. In some ways, a rural-serving identity made institutions more flexible and better able to adjust their advising and curricular structures to comply with the mandates of SB 1720. Either because of physical proximity of office space or a culture of relationships, campus personnel were able to collaborate with one another and with community members to implement the best version of the bill they could for students. Moreover, the culture of rural-serving institutions around providing personalized attention, in-person advising, and increased academic supports helped students succeed following SB 1720 in ways they wouldn’t have been able to on urban campuses.

In other ways, rural-serving institutions lacked the resources necessary to adequately support students and campus personnel as they transitioned toward a new way of doing DE. Rural colleges nationwide have long struggled with limited funding and leveraging economies of scale (Hardy and Katsinas 2007; Pennington et al. 2006). Because SB 1720 required expensive changes and a good deal of individualization for students, these problems were described as being made worse. For instance, on many of the campuses DE enrollment was too limited for colleges to be able offer students the choice between accelerated and corequisite courses. Taking a perspective contrary to our participants, we question if SB 1720 has the potential to improve class enrollment issues by consolidating students in one college-level course, rather than spreading them across three levels of DE coursework. Returning to the discussion of limited capacity, the colleges and their surrounding communities also lacked the ICT infrastructure necessary for successful, widespread implementation of computer-based courses. Even still, campus personnel reported finding a way to accomplish more for students, even without increased funding or support from the state.

### Contributions and Future Research

Academic life on rural-serving college campuses has largely been ignored by scholars (Sipple and Brent 2009). Although individual rural-serving institutions enroll a small number of students relative to urban institutions, they collectively serve a large number of students nationwide (Hardy and Katsinas 2007) and play critical roles in their communities as engines for educational and economic development (Cavan 1995; Crookston and Hooks 2012; Siegfried et al. 2007). In discussions about DE reform, particularly in Florida, rural scholar Andrew Koricich notes, “What’s glaringly missing is talk about place and locale” (Smith 2019). Our research directly addresses his critique by demonstrating exactly how SB 1720 was implemented at rural-serving colleges according to campus personnel and students.

More broadly, our work serves to remind policy makers of the importance of considering rural-serving community colleges when passing legislation (Hardy and Katsinas 2007). This consideration might occur during the creation phase of policy, or take the form of accommodations and exemptions after it has been passed. Although accommodations and exemptions may not always be the most standardized or efficient course of action, it is important to acknowledge that the health and success of rural-serving institutions have many implications for the communities surrounding them. Looking ahead, Florida educators can anticipate a number of upcoming education initiatives, like math pathways reform. Our findings elevate the need to consider what exemptions might be made to ensure that new mandates are successful in the panhandle and peninsula alike.

Preliminary research indicates that this bill has benefited certain student groups (Hu et al. 2016b), but it remains to be seen whether impacts are equally distributed between rural and urban areas (Smith 2019). The findings of this study suggest that there are differences—likely positive and negative—that require future exploration. Opportunities to study the effects of this bill on institutions with unique geographic contexts and identities, such as urban or suburban colleges, Military-Friendly Colleges, and Minority-Serving Institutions exist. In each instance, institutional culture will have dictated implementation of reform. Comparing the findings presented here with quantitative measures of student success at the rural-serving colleges in question can also determine how persistence and degree attainment have changed following the passage of SB 1720.
